# Treatment of experimental periodontal disease by laser therapy in simvastatin-modified rats

**DOI:** 10.1590/1678-7757-2016-0467

**Published:** 2017

**Authors:** Andressa Araújo SWERTS, Bianca Fernanda Espósito SANTOS, Simone Ribeiro BRUZADELLI, Maísa Ribeiro Pereira Lima BRIGAGÃO, Daniela Coelho de LIMA, Leandro Araújo FERNANDES

**Affiliations:** 1 Universidade Federal de Alfenas, Faculdade de Odontologia, Departamento de Clínica e Cirurgia, Alfenas, MG, Brasil.; 2 Universidade Federal de Alfenas, Faculdade de Odontologia, Departamento de Bioquímica, Alfenas, MG, Brasil.

**Keywords:** Alveolar bone, Laser therapy, Oxidative stress, Periodontal diseases, Simvastatin

## Abstract

Low intensity laser can be used as a promising alternative in the treatment of periodontal disease.

**Objective:**

The aim of this study was to evaluate low-level laser therapy (LLLT) as an adjuvant treatment for scaling and root planing (SRP) for the treatment of induced periodontitis in simvastatin-modified rats.

**Material and Methods:**

A total of 180 rats were evenly divided into two groups: Veh – receiving oral administration of polyethylene glycol (vehicle); S – receiving oral administration of Simvastatin. Periodontal disease was induced in both groups at the first mandibular molar. After seven days, the ligature was removed and the animals were divided into subgroups according to the following local treatments: NT – no treatment; SRP – scaling and root planing and irrigation with saline solution; and LLLT ¬– SRP and laser irradiation (660 nm; 0.03 W; 4 J). Ten animals in each subgroup/local treatment were euthanized at 7, 15 and 30 days. Samples of gingival tissue were processed to analyze the tissue oxidative damage and radiographic analysis. Levels of oxidative stress were analyzed by the expressions of Tripeptideglutathione (TG), Malondialdehyde (MDA) and Carbonylated Proteins (CP).

**Results:**

The animals in S group had higher levels of TG and lower levels of MDA and CP compared with Veh group (p<0.05). Radiographically, in the intragroup analysis Veh and S, LLLT showed lower bone loss (BL) compared with NT and SRP, in all experimental periods (p<0.01). In addition, a lower BL was observed for the animals of Veh group treated with LLLT compared with treatment SRP in the S group, in all experimental periods.

**Conclusion:**

Within the limits of this study, we can conclude that LLLT was effective as adjuvant treatment for SRP protecting against the occurrence of oxidative tissue damages as well as for reducing alveolar bone loss in experimentally induced periodontitis simvastatin-modified rats.

## Introduction

In inflammation, neutrophils are the first cells to be activated to defend the body together with macrophages^27^. Those are chemotaxically attracted by secretory cells, bacteria and other foreign bodies to inflammation areas^27^. On this site, neutrophils phagocyte the microorganisms, covered or not with complement or specific antibodies, which are killed by cytotoxic proteins derived from cytoplasmic granules and by oxygen and nitrogen reactive species such as superoxide anion^9^, hydrogen peroxide^28^, hydroxyl radical^12^ and peroxynitrite^21^.

There is evidence of a more aggressive destruction of tooth support tissues with elevated levels of oxidative stress markers during the development of periodontal disease (PD)^24^. Among the oxidants, the superoxide anion^9^, in periodontal tissues, can be involved in signaling of induction of bone resorption; the hydroxyl radical^12^ is extremely reactive and can damage important biomolecules, such as proteins, lipids and nucleic acids, whereas hydrogen peroxide is able to cross membranes, damaging adjacent cells and increasing the oxidative cascade^28^. Thus, most studies demonstrate that periodontitis is associated with increased lipid peroxidation^29^ and increased protein carbonyls^9^ as well as decreased antioxidants, such as reduced glutathione (GSH)^11,29^In addition, there are evidences of decreased oxidative injuries and changes in antioxidant system following periodontal treatment^19^.

The treatment of PD is based on the elimination of pathogenic subgingival microbiota by scaling and root planing (SRP)^24^. However, mechanical therapy used alone may be defective in the elimination of pathogenic bacteria, since they are located within soft and hard tissues or in areas that are inaccessible to periodontal instruments^7^. In addition, an important component of individuals themselves can lead to tissue destruction observed in the periodontitis. Therefore, therapeutic strategies performing the pharmacological modulation of host response have emerged as a new therapeutic approach^13^.

Simvastatin is an inhibitor of the 3-hydroxy-3-methyl-glutaryl-coenzyme A (HMG - CoA reductase) enzyme, which is responsible for the synthesis of cholesterol and therefore it is widely used for the systemic treatment of diseases related to hypercholesterolemia. This drug also has anti-inflammatory, immunomodulator, antioxidant, and angiogenic effects^6,25^, and it also promotes increased osteoblast formation^10,20^. Such properties offer great potential for statins to modify the course of chronic inflammatory diseases such as periodontitis^26^.

In addition to drug therapy, the use of low intensity lasers associated with scaling and root planing for the local treatment of periodontal disease has been reported^7^. On the other hand, there are no studies evaluating the local effects of low-level laser therapy (LLLT) associated with the systemic effects of statins. Thus, the aim of this study was to evaluate LLLT as an adjuvant treatment for scaling and root planing (SRP) in the treatment of induced periodontitis in simvastatin-modified rats.

## Material and methods

### Animals

This study was conducted on 180 adult male Wistar rats (200–250 g). The animals were kept in plastic cages with access to food and water *ad libitum*. Prior to surgical procedures, all animals were allowed to acclimatize to the laboratory environment for a period of five days. All protocols described below were approved by the Ethics Committee on the Use of Animals (CEUA), following the standards adopted by the Brazilian College of Animal Experimentation (COBEA), under protocol 472/2012.

### Study design

Animals were numbered and randomly divided into two groups: Veh group (n=90) received Polyethylene Glycol 400 (All Chemistry; São Paulo, SP, Brazil) at 0.5 mg/kg body weight (vehicle), and S group (n=90) received Simvastatin (Medley; Campinas, SP, Brazil) at 0.5 mg/kg body weight orally^18^. Administrations were daily performed in a single dose, starting 24 h before induction of PD and maintained until the end of the respective periods of euthanasia. Animals were weekly weighed throughout the experimental period to maintain the doses.

Simvastatin preparation was performed by diluting 400 mg of the drug in 400 mlL of Polyethylene Glycol to reach a final concentration of 1 mg/mL (Moss Manipulation Pharmacy; Alfenas, MG, Brazil).

### Induction of experimental periodontal disease

General anesthesia was induced by administering ketamine (0.4 mL/kg) (Fort Dodge Animal Health Ltda; Campinas, SP, Brazil) together with xylazine (0.2 mL/kg) (Coopers; São Paulo, SP, Brazil) via intramuscular injection. The mandibular left first molar from each animal in both Veh and S groups was selected to receive a cotton ligature No. 10 (Coats; São Paulo, SP, Brazil) in a submarginal position to induce experimental periodontitis^2^.

### Local treatment

After seven days of periodontal disease experimental induction, mandibular ligature was removed from the left first molar of all animals in groups Veh and S. The animals were divided into subgroups according to the local treatments (performed only once): NT – no treatment; SRP – scaling and root planing and irrigation with saline solution; and LLLT – SRP and laser irradiation.

Left molars were subjected to SRP with manual #1–2 micro mini five curettes (Hu-Friedy; Chicago, IL, USA) through 10 distal–mesial traction movements in both buccal and lingual aspects. The furcation and interproximal areas were scaled with the same curettes through cervical-occlusal traction movements. The entire SRP procedure was performed by the same experienced operator. The saline solutions were slowly deposited within the periodontal pocket using syringe (1 mL) and insulin needle (13 mm x 0.04 mm) (Becton Dickinson; Curitiba, PR, Brazil).

The laser used in this study was gallium–aluminium–arsenide (GaAlAs) (Kondortech Equipment Ltd; São Carlos, SP, Brazil), with a wavelength of 660 nm and a spot size of 0.07 cm^2^. After 1 min of saline solution application, LLLT was applied to three equidistant points at each buccal and lingual aspect of the mandibular first molar in contact with the tissue. The therapeutic laser was released with a power of 0.03 W for 133 s/point, a power density of 0.428 W/cm^2^, and energy of 4 J/point (57.14 J/cm^2^/point)^7^.

### Experimental periods

Ten animals from each experimental subgroup/local treatment were euthanized by exsanguination at 7, 15 and 30 days following local treatments. The jaws were removed and, in order to analyze the levels of oxidative stress, collections of the inserted and marginal gums of the buccal faces of the first left lower molars were performed. Then, the jaws were split in the middle and fixed in 10% buffered formalin for at least 48 h for later radiographic analysis of the left side.

### Preparation of gingival samples

Gingival tissue samples were homogenized with phosphate buffer (3 mL, pH 6.5). The homogenate was centrifuged at 3500 rpm for 10 min. The supernatant was separated into aliquots for subsequent biochemical analysis.

### Determination of the total protein concentration

Protein concentrations were determined in all samples of gingival homogenates by the method of Bradford^4^, using bovine serum albumin (BSA) as a standard calibration curve. The total protein concentration is routinely determined for normalization of biochemical results. Thus, the expression results in the mass in mg of tripeptideglutathione (TG), malondialdehyde (MDA) and carbonylated proteins (CP) *per* mg protein (units).

### Determination of tripeptideglutathione (TG)

For verification of TG in the gingival tissue of rats alkylated derivatives of mBBr were separated by HPLC in C-18 column (Shim-pack VP-ODS, 4.6 mm x 25 cm, 5 µm, connected in series with C-18 precolumn model Shim-pack GVP-ODS, 4.6 mm x 10 mm), equilibrated with buffer A (14.2% ethanol and 0.25% acetic acid m/v, 1 mL/min). Samples of tissue suspensions derivatized with mBBr were injected and dilution (buffer A) was performed for 30 min. Then, the column was washed with buffer B (90% methanol and 0.25% acetic acid) for 8 min and re-equilibrated with buffer A. Sulfosalicylic acid (internal reference standard), cysteine and reduced glutathione were determined by comparing the retention time of authentic standards (10-200 nmol) using a fluorescence detector (model RF-10AXL) and λexc=394 nm and λemi=490 nm (ε=20 mM^-1^.cm^-1^), and the quantification was performed by counting the units in the respective areas (Software “LC-Solution Multi”)^14^.

### Determination of malondialdehyde (MDA)

The MDA verification through homogenized gum samples was performed by adding 250 μL of 1.22 M phosphoric acid, 450 μL of Milli-Q Water and 250 μL of TBA reagent. Thereafter, aliquots are stirred for 30 s and the reaction is incubated for 1 h in water bath at 95°C and cooled in ice bath at 4°C. After this process, 360 µL of methanol (HPLC grade) and 40 µL of 1M NaOH are added to a 200 µL sample, in order to neutralize the solutes and precipitate proteins before injecting into the HPLC column, using a fluorescence detector (model RF-10AXL) and λexc=515 nm and λemi=553 nm^14^.

### Determination of carbonylated proteins (CP)

In order to determine these CP, 500 µL of the aliquots in phosphate buffer (pH 7.2) may be used, adding 500 µL of 2.4-dinitrophenylhydrazine solution (10 mμ). After that, incubate for 1 h and then apply (drops) 500 µL of 20% TCA solution until complete precipitation. Add 500 µL of Ethanol/Ethyl-Acetate solution and centrifuge at 2000 rpm for 10 min. Discard the supernatant and re-add 500 µL of ethanol/ethyl-acetate solution and centrifuge it. Then, discard the supernatant and dissolve the precipitate with 1000 µL of 6M guanidine, which should be homogenized. The rate of change in absorbance was spectrophotometrically measured at 370 nm^14^.

### Radiograph and digital analysis

After fixation of the hemimandibules (HMs) in 10% buffered formalin, the left side was submitted to X-ray procedure.

HMs were positioned on a table with the vestibular surfaces facing the radiographic film (Eastman Kodak Company; Rochester, Nova York, EUA), in such a way that the right side stayed at the bottom, and the left side stayed at the top.

Standardization of radiographs was obtained as follows:

Use of an X-ray device Pampas - E (CDK X-ray Equipment; Diadema, SP, Brazil), with electric system of 65 kvp, 10 mA;

Central X-ray beam perpendicular to the film-object plane, at a 90-degree angle in relation to the surface of the optical plate;

Focal length of 30 cm;

Exposure time of 0.8 seconds.

Radiographs were developed using solutions from Kodak developer and fixer, using the climate-weather development method.

They were scanned and the images were analyzed with the Imagelab software (Softium; São Paulo, SP, Brazil) using the tool distance and angle of measurement. With this feature, it was measured the distance from the cementoenamel junction to the alveolar bone crest on the mesial surface of the first left lower molars by drawing a line, and these measurements were recorded in millimeters (mm). The mouse was positioned on the region corresponding to the cementoenamel junction. By left-clicking and dragging the mouse down to the level of alveolar bone crest the software automatically measured the distance.

### Statistical analysis

Statistical analysis of oxidative stress and radiographic data was performed by BioEstat 3.0 software (Sonopress; Manaus, AM, Brazil). The hypothesis of absence of a statistically significant difference in the data obtained in the region of the mandibular first molars between different groups, subgroups/treatments and periods in the teeth with induced periodontitis was tested. After the analysis of the normality of the data by the Shapiro-Wilk test, multiple comparisons among the variables were performed by two-way analysis of variance ANOVA with supplementation by the Tukey test, with p<0.05 (oxidative stress data) and by the Bonferroni test, with p<0.01 (radiographic data).

## Results

Expression of TG in gingival samples

In the intragroup comparison (Veh and S), TG levels increased significantly (p<0.05) between the experimental periods in all local treatments. Regarding local treatments, LLLT showed a significant increase (p<0.05) in GSH compared with NT and SRP at 7 and 30 days.

In the comparison between the groups (Veh and S), among the same local treatments, TG levels in the S group were significantly higher than those in the Veh group (p<0.05), for NT, SRP and LLLT in all experimental periods (Table 1).

**Table 1:** Means and standard deviations (M±SD) expression in TG units in the gingival tissue of the first lower left molar, according to each group, local treatment and period

Group	Veh		
Periods	7 days	15 days	30 days
Treatments			
NT	5.02±0.24#*´°	10.99±0.30#´°	22.17±1.21#*´°
SRP	8.52±1.00 #*´°	15.01±0.30#´°	40.76±1.98#*´°
LLLT	10.13±1.13#*´°	16.07±1.64#´°	52.49±0.58#*´

**Group**		**S**	
**Periods**	**7 days**	**15 days**	**30 days**
**Treatments**			

NT	14.15±0.34#*´°	23.11±1.67#´°	43.87±0.56#*´°
SRP	17.43±0.02#*´°	26.79±1.02#´°	54.97±1.02#*´°
LLLT	23.29±0.17#*´°	24.32±1.09#´°	64.98±1.12#*´°
N	60	60	60

#Difference among same groups and local treatments (ANOVA and p<0.05)

*Difference between local treatments, same group and period (ANOVA and Tukey, p<0.05)

´Difference between groups, same local treatment and period (ANOVA and Tukey, p<0.05)

°Difference between groups and local treatments, same period (ANOVA and Tukey, p<0.05)

### Expression of MDA in gingival samples

In the intragroup comparison, levels of MDA in LLLT decreased significantly (p<0.05) compared with NT, in the S group in all experimental periods, and in the Veh group at 7 and 30 days.

In the comparison between the groups (Veh and S), among the same local treatments, MDA levels in S group were significantly lower than those in Veh group (p<0.05), for NT, SRP and LLLT at 7 days (Table 2).

**Table 2:** Means and standard deviations (M±SD) expression in MDA units in the gingival tissue of the first lower left molar, according to each group, local treatment and period

Group	Veh		
Periods	7 days	15 days	30 days
Treatments			
NT	0.18±0.06´°	0.24±0.12°	0.30±0.03°
SRP	0.28±0.12´°	0.40±0.09	0.62±0.13*
LLLT	0.48±0.32*´°	0.47±0.43*	0.34±0.17°

**Group**	**S**		
**Periods**	**7 days**	**15 days**	**30 days**
**Treatments**			

NT	0.34±0.07´°	0.36±0.16	0.44±0.08°
SRP	0.53±0.22´°	0.38±0.65	0.61±0.73*°
LLLT	0.72±0.54*´°	0.44±0.33*°	0.41±0.98°
N	60	60	60

#Difference among same groups and local treatments (ANOVA and p<0.05)

*Difference between local treatments, same group and period (ANOVA and Tukey, p<0.05)

´Difference between groups, same local treatment and period (ANOVA and Tukey, p<0.05)

°Difference between groups and local treatments, same period (ANOVA and Tukey, p<0.05)

### Expression of CP in gingival samples

In the intragroup comparison (Veh and S), levels of CP in LLLT decreased significantly (p<0.05) in relation to NT in all the experimental periods.

In the comparison between Veh and S groups, among the same local treatments, CP levels in the S group were significantly lower than those in the Veh group (p<0.05), for NT, SRP and LLLT at 7 days. In addition, CP levels in the Veh group, treated by LLLT, were significantly lower than those in the S group (p<0.05), which did not receive local treatment (NT) at 7 and 15 days (Table 3).

**Table 3:** Means and standard deviations (M±SD) expression in CP units in the gingival tissue of the first lower left molar, according to each group, local treatment and period

Group	Veh		
Periods	7 days	15 days	30 days
Treatments			
NT	13.23±0.31+*´°	8.45±1.56+*	7.89±1.95*°
SRP	7.02±0.31+*´°	2.39±1.13+*°	3.92±0.06+*°
LLLT	4.71±0.23+*´°	1.99±0.94+*°	2.37±0.18+*°

**Group**	**S**		
**Periods**	**7 days**	**15 days**	**30 days**
**Treatments**			

NT	9.78±.3+*´°	7.99±11.3+*°	5.21±0.04+*°
SRP	1.29±1.16+*´°	4.80±1.12+*°	3.92±1.00+*°
LLLT	1.57±0.95+*´°	3.41±1.04+*°	1.09±0.32+*°
N	60	60	60

#Difference among same groups and local treatments (ANOVA and p<0.05)

*Difference between local treatments, same group and period (ANOVA and Tukey, p<0.05)

´Difference between groups, same local treatment and period (ANOVA and Tukey, p<0.05)

°Difference between groups and local treatments, same period (ANOVA and Tukey, p<0.05)

### Radiographic analysis

In the intragroup analysis between Veh and S, LLLT showed lower BL compared with NT and SRP, in all experimental periods (p<0.01).

In the intergroup analysis between the same local treatments, the SRP presented a lower BL (p<0.01) in the animals of the S group (1.61±0.45 mm) compared with those in the Veh group (1.88±0.31 mm) in the 7-day period.

**Figure 1:**
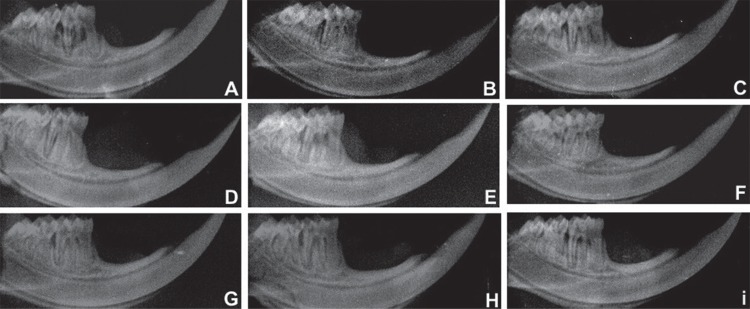
Radiographic images of the hemimandibules of animals of the Veh group – A: NT 7 days; B: NT 15 days; C: NT 30 days; D: SRP 7 days; E: SRP 15 days; F: SRP 30 days; G: LLLT 7 days; H: LLLT 15 days; and I: LLLT 30 days

**Figure 2:**
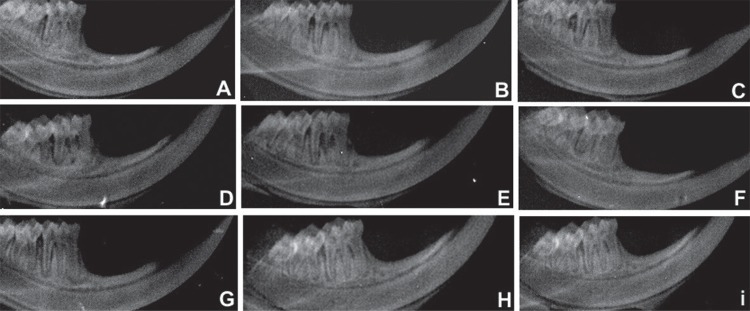
Radiographic images of the hemimandibules of animals of the S group – A: NT 7 days; B: NT 15 days; C: NT 30 days; D: SRP 7 days; E: SRP 15 days; F: SRP 30 days; G: LLLT 7 days; H: LLLT 15 days; and I: LLLT 30 days

In the S group, LLLT values (0.57± mm, 0.50±0.55 mm, 0.47±0.88 mm) showed lower BL (p<0.01) compared with animals of the Veh group treated with SRP (1.88±0.31 mm, 1.83±1.06 mm, 1.51±0.19 mm) in all experimental periods. In addition, a lower BL was observed for the animals of the Veh group treated with LLLT (0.63±1.17 mm; 0.58±0.93 mm; 0.49±1.34 mm) compared with SRP treatment in the S group (1.61±0.45 mm; 1.49±0.56 mm; 1.36±1.31 mm), in all experimental periods (Table 4, Figure 1 and Figure 2).

**Table 4:** Means and standard deviations (M±SD) of the distances between the cementoenamel junction and alveolar bone crest (mm) on the mesial surface of the first lower left molar, according to each group, local treatment and period

Group		Veh	
Periods	7 days	15 days	30 days
Treatments			
NT	2.97±0.21+*°	2.53±0.34+*°	2.06±0.13+*°
SRP	1.88±0.31+*´°	1.83±1.06*°	1.51±0.19+*°
LLLT	0.63±1.17*°	0.58±0.93*°	0.49±1.34*°

**Group**		**S**	
**Periods**	**7 days**	**15 days**	**30 days**
**Treatments**			

NT	2.65±0.33+*°	2.37±0.43+*°	1.74±0.97+*°
SRP	1.61±0.45*´°	1.49±0.56*°	1.36±1.31*°
LLLT	0.57±0.78*°	0.50±0.55*°	0.47±0.88*°
N	60	60	60

#Difference among periods, same groups and local treatments (ANOVA and Bonferroni, p<0.01)

*Difference between local treatments, same group and period (ANOVA and Bonferroni, p<0.01)

´Difference between groups, same local treatment and period (ANOVA and Bonferroni, p<0.01)

°Difference between groups and local treatments, same period (ANOVA and Bonferroni, p<0.01)

## Discussion

Periodontal disease is a chronic inflammatory process characterized by gingival bleeding, formation of periodontal pockets and destruction of periodontal supporting tissues, through the release of lipopolysaccharide and proteases^27^ of bacteria present in the dental biofilm, a key etiological factor of this change. This tissue inflammation is associated with increased release of oxygen reactive species (ORS) by neutrophils, and also with the activation of several inflammatory mediators, such as interleukins (IL-1β, 6 and 8) and tumor necrosis factor (TNF-α)^11^, which promotes the imbalance in bone homeostasis, resulting in destruction of alveolar bone tissue, increased activity of matrix metalloproteinases and connective tissue degradation^17^, due to exacerbated immune response and localized osteoclastogenesis^12^.

Periodontal treatment is used to paralyze the destruction of the supporting tissues of the teeth in order to avoid their loss^5^. However, there are cases where isolated periodontal mechanical therapy is ineffective, suggesting that systemic factors, not yet understood, could interfere with the development of the disease^22^. Thus, the use of antioxidants could be an adjuvant therapy to conventional treatment^17^.

Simvastatin is a drug with hypolipidemic function, but it stands out for other minor effects, including anti-inflammatory^25^, immunomodulatory, and antioxidant effects, and the promotion of angiogenesis and increased differentiation of osteoblasts, inducing bone formation^10,20^. Such properties offer great potential for statin to modify the course of chronic inflammatory diseases^26^ such as chronic periodontitis.

Ligature-induced alveolar bone loss may occur due to abnormal activation of the host’s immune system^27^. This will result in an imbalance, leading to an excessive production of oxidants and inhibiting the formation of antioxidants, which will cause the development of oxidative stress^11^. The association between systemic oxidative stress and periodontal disease in human and animal studies has been described in a review of the literature by Tomofuji, et al.^28^ (2011). This author mentioned that there is a correlation between the production of oxidants in sites with periodontal disease and the development of lesions in various organs of the body.

In animals, the association between ORS/nitrogen reactive species (NRS) in sites of induced periodontitis is well established. With the use of an experimental model induced by topical application of lipopolysaccharide and specific proteases to the gums, Tomofuji, et al.^28^ (2011) showed a clear correlation between the severity of periodontal disease and oxidative lesions in the liver tissues and descending aorta. This oxidative stress can be evaluated in several ways such as by measuring the ORS levels, damages to nucleic acids, proteins and lipids, and detecting the levels of antioxidants^3^.

In this context, the proposed experimental model evaluated the concentration of TG, an endogenous antioxidant, and our results demonstrated that in both groups (Veh and S) NT showed significantly reduced levels compared with SRP and LLLT. This fact demonstrated that the development of oxidative damage occurred as well as in another study described in the literature using experimental periodontitis in rats^11^.

Another widely used method for determining the occurrence of oxidative damage mediated by ORS in tissues is the measurement of MDA^3^. Studies have demonstrated an association between periodontitis and increased MDA in samples of gingival fluid, saliva and gingival tissue^11,28^. In the intragroup comparison we observed that levels of MDA in NT were significantly higher (p<0.05) compared with LLLT, in the S group in all experimental periods, and in the Veh group at 7 and 30 days. These results demonstrate that MDA is a good marker of oxidative stress. Our results corroborate other studies^11,23^, which report that lipid peroxidation is a very common example of oxidative stress in induced periodontitis and that its increase plays an important role in the progression of periodontal destruction^3^.

The CP analysis is another way to verify the presence of oxidative tissue damage with changes mediated by inflammatory processes. In this model we observed a significant decrease in CP in the LLLT in the S group compared with NT and SRP in Veh group (p<0.05). This is probably due to the anti-inflammatory effects of LLLT and simvastatin. A study carried out in 2003^16^ reinforced this hypothesis, since it demonstrated that simvastatin was able to inhibit the secretion of matrix metalloproteinases. Thus, they could reduce the inflammatory response, providing protection against the destruction of periodontal tissue. In humans, some studies have established that chronic periodontitis is directly correlated with the high occurrence of oxidative damage to proteins, determined by the measurement of serum CP as well as the increase in total oxidant status and lipid peroxidation products, quantified as MDA^15,30^. Whereas regarding LLLT, it was observed that it inhibited the production of inflammatory mediators^1^.

In the intragroup analysis between Veh and S groups, LLLT showed lower BL compared with NT and SRP in all experimental periods (p<0.01). These results demonstrate that SRP was not effective in controlling bone loss in the furcation areas of animals. Clinically, it is proved that SRP with hand tools provides the best results for the treatment of periodontal disease. However, several factors may limit the success of SRP such as root concavities, dental crowding, deep areas, and areas of bifurcation that hinder the access of hand tools in the periodontal pocket. Due to these limiting anatomic factors, therapies to support conventional treatment have been proposed^15^.

Therefore, in this study simvastatin was chosen, since statins have different effects on bone, such as increased bone formation, whereas lovastatin and pravastatin have a smaller effect than simvastatin, atorvastatin and cerivastatin^10^. In addition, it stands out for acting in important events during an exacerbated inflammatory response. In the intergroup analysis between the same local treatments, the SRP presented a lower BL (p<0.01) in the animals of the S group compared with those in Veh group in the 7-day period. In S group, LLLT values showed lower BL (p<0.01) compared with animals of Veh group treated with SRP in all experimental periods. This result is in agreement with other studies^3,10,15^. According to Luan, et al.^16^ (2003) statins decrease the production of many pro-inflammatory cytokines and have also been described as promoting decreased secretion of MMP-1 (matrix metalloproteinase - 1), MMP-2, MMP-3 and MMP-9 *in vitro*. Thus, they could reduce strong immune response, protecting against the destruction of periodontal tissue.

In addition, a lower BL was observed for the animals of the Veh group treated with LLLT compared with SRP treatment in the S group, in all experimental periods, confirming previous studies^5,7^ that demonstrate a better outcome of periodontal treatment with this combination, by stimulating bone formation^7,8,22^. These studies have reported that the use of this light source inhibits the production of inflammatory mediators by cells of the periodontal ligament, promotes cell chemotaxis, and promotes local angiogenesis and vasodilation, and therefore there could be an increase in tissue oxygen diffusion, promoting the repair process, because the secretion of collagen by fibroblasts in the extracellular space only occurs in the presence of high rates of oxygen pressure^1^. However, in a meta-analysis study^23^, the results showed no difference when comparing the treatment of periodontal disease through SRP or in combination with lasers. These conflicting results may be due to methodological differences, mainly in relation to the protocols of laser used and to the different irradiation parameters used.

Among the limitations of the study, we can mention the fact of being carried out in animals, and it is not prudent to extrapolate the results to the human species, for further studies in the literature would be necessary.

## Conclusion

Within the limits of this study, we can conclude that LLLT was effective as adjuvant treatment for SRP protecting against the occurrence of oxidative tissue damages as well as reducing alveolar bone loss in experimentally induced periodontitis simvastatin-modified rats.
